# Looking to the Future: Prospective Life Cycle Assessment of Emerging Technologies

**DOI:** 10.1002/chem.202500304

**Published:** 2025-04-03

**Authors:** Alessandro Marson, Alberto Benozzi, Alessandro Manzardo

**Affiliations:** ^1^ CESQA (Quality and Environmental Research Centre), Department of Civil, Environmental and Architectural Engineering University of PadovaVia Marzolo 9 Padova Italy

**Keywords:** anticipatory life cycle assessment, life cycle assessment, prospective life cycle assessment, scenario analysis, upscaling

## Abstract

Emerging technologies are expected to contribute to sustainable development, but assessing their future environmental impacts compared to current technologies remains challenging. Prospective life cycle assessment (pLCA) provides a systematic approach to evaluating emerging technologies throughout their life cycle and projecting impacts at future industrial scales. This review aims to identify critical aspects of pLCA methodologies and examine their implementation in recent case studies. Key aspects are highlighted: assessment of initial technology maturity, upscaling methods to model data at higher technology readiness levels, and development of future scenarios to contextualize the scaled‐up systems. Specific upscaling techniques such as process simulation, engineering calculations and technology learning curves are discussed. Approaches to generating future scenarios in line with integrated assessment models and common socio‐economic pathways are also analyzed. An in‐depth literature review covering 2021–2024 publications categorizes 79 articles into literature reviews, methodological papers and pLCA case studies. The identified critical issues serve as a benchmark to assess the completeness and robustness of 20 case studies. Notable contributions, limitations and the need for comprehensive guidelines that integrate scenario development with upscaling methods are highlighted. This review provides valuable insights for researchers conducting pLCA studies of emerging technologies and materials.

## Graphical abstract

This review explores prospective life cycle assessment (pLCA), an emerging methodology for evaluating the environmental impacts of technologies still in development. Key aspects are identified, including assessing technology maturity levels, upscaling methods, and generating future scenarios. Recent case studies are analyzed to guide researchers in implementing robust pLCA frameworks for emerging technologies.

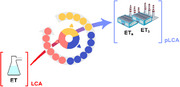

## 1 Introduction

Emerging technologies are expected to make a significant contribution to sustainable developments;^[^ ^1^ ^]^ however, it remains unclear whether these technologies will reduce environmental impacts in the future compared to already established commercial technologies.^[^ ^2^ ^]^ This uncertainty underscores the importance of carefully assessing the potential environmental impact of new technologies from the early stages of their development.

Life cycle assessment (LCA) is recognized as a valuable tool for evaluating the environmental impacts of services and products.^[^ ^3^ ^]^ When applied to emerging technologies, LCA offers a systematic approach to analyze the entire life cycle of a technology, from its conception to final disposal. This comprehensive analysis is particularly crucial in the context of technological innovation and sustainability.

The application of LCA to emerging technologies becomes especially relevant in light of the “design paradox”, also known as the Collingridge dilemma.^[^ ^4^ ^]^ This concept states that the ability to change the design of products and processes is inversely proportional to their knowledge and market diffusion. In other words, the more developed and widespread a technology becomes, the more difficult it is to make significant changes to its design. This consideration highlights the critical importance of integrating LCA with emerging technologies at an early stage of development, before they reach the market. Early life cycle analysis can lead to improved eco‐design solutions, allowing for the identification and mitigation of potential negative environmental impacts when design modifications are still relatively easy to implement.

Although the primary goal of conducting an LCA is often to provide information that supports decision‐makers and designers with a future‐oriented perspective, most LCA studies are retrospective in nature and do not explicitly consider potential future effects.^[^ ^5^ ^]^ This limitation becomes particularly problematic when dealing with emerging technologies, as their performances evolves over time due to increasing technological maturity and a deeper understanding of the process involved.^[^ ^1^, ^3^ ^]^ Consequently, directly applying retrospective LCA methodologies to emerging technologies can result in weaknesses and inconsistencies, as the data may rely on processes with varying levels of technological readiness. In other terms, larger or more advanced industrial processes may achieve lower environmental impacts per unit of output compared to current emerging systems, benefiting from both accumulated learning effects and the adoption of more efficient equipment over time.^[^ ^1^, ^3^ ^]^ This gap between emerging and mature systems hinders the accuracy of future‐oriented comparisons.^[^ ^6^ ^]^ To address these challenges and enable more meaningful comparisons between emerging and mature technologies, a new set of methodologies has been integrated into LCA procedures. These methodologies, collectively known as prospective LCA (pLCA), allow for consistent and appropriate comparisons within a predefined future time horizon. pLCA approaches are designed to account for the potential evolution of technologies and their environmental impacts over time.

The innovative aspect of pLCA, also referred to as “Ex‐Ante” or “Anticipatory” LCA,^[^ ^7^ ^]^ lies in its ability to project emerging technological systems into a more distant future ($t_{f}$), compared to the current time. This approach involves scaling up all potential alternative emerging technologies to the same technological readiness level (TRL).^[^ ^2^ ^]^ Through this approach, pLCA establishes a standardized basis for comparison, facilitating equitable assessment of technologies at varying stages of development. This methodology ensures that innovations can be evaluated on comparable grounds, regardless of their maturity level.

Performing this type of future‐oriented projection addresses more radical technological changes over longer time frames, rather than incremental changes close in time.^[^ ^4^ ^]^ This broader perspective is crucial for understanding the long‐term environmental implications of emerging technologies and their potential to contribute to sustainable development goals.

pLCA facilitates an equitable comparison between nascent and established technologies by evaluating them at equivalent technology readiness levels (TRLs). This methodology simulates the prospective environmental impacts of emerging technologies in their anticipated saturation phase, juxtaposed with current mature solutions.^[^ ^5^ ^]^ Consequently, this approach enhances informed decision‐making for long‐term technological development and implementation.

Despite its benefits, pLCA faces challenges into comparability, data availability and quality, and uncertainties.^[^ ^2^ ^]^ These issues hinder the creation of standardized guidelines, resulting in multiple approaches in literature.^[^ ^8^ ^]^ The absence of formal guidelines is particularly problematic because the comparability of results can be significantly influenced by the choice of pLCA methodology rather than the inherent characteristics of the emerging technology being assessed. This methodological dependency introduces a level of uncertainty and potential bias in the results (typical of LCA^[^ ^9^ ^]^), which can undermine the credibility and usefulness of pLCA studies.

The absence of a standardized pLCA methodology and a comprehensive review of recent publications has led to limited awareness and adoption of this assessment tool among key stakeholders, including scientists developing new materials and technologies, particularly at the laboratory scale. Furthermore, we present a state‐of‐the‐art review on managing the most critical aspects of pLCA.

This study synthesizes recent literature, encompassing reviews and case studies published post‐2021, which were not addressed in earlier surveys. Our approach provides a current and thorough examination of pLCA methodologies and their implementations. The resulting analysis offers valuable insights for researchers and scientists engaged in advanced technological and material innovations, including a step‐by‐step guideline for pLCA application.

## 2 Prospective Life Cycle Assessment

Before exploring prospective life cycle assessment (pLCA), it is essential to outline the main variants of the LCA methodology. This framework will help readers understand the diversity of LCA approaches and their applications.

A key debate in LCA terminology involves attributional LCA (ALCA) and consequential LCA (CLCA). ALCA describes the environmental impacts of a system as it exists (or is projected to exist), while CLCA evaluates the consequences of decisions, such as market shifts or policy changes. Although pLCA is often linked to CLCA, prospective studies can align with either framework depending on their goal—modeling a static future system (ALCA) or dynamic responses to changes (CLCA).^[^ ^4^ ^]^

Beyond ALCA/CLCA, newer LCA approaches have emerged outside ISO standards. These include Explorative LCA (XLCA), a conceptual umbrella term for methods that analyze hypothetical or future‐oriented systems. Examples from niche literature include backcasting LCA (BLCA), scenario‐based dynamic LCA (SLCA), and integrated LCA (ILCA).^[^ ^10^ ^]^ Within XLCA, prospective LCA (pLCA) focuses on assessing products or technologies projected into a defined future timeframe.^[^ ^7^ ^]^ Arvidsson et al. define pLCA as an evaluation of emerging technologies modeled at a more advanced development stage (e.g., transitioning from lab‐scale to industrial production).^[^ ^4^ ^]^

However, broader definitions now emphasize pLCA as “modeling a product system at a future point in time relative to the study's execution”,^[^ ^7^ ^]^ prioritizing temporal positionality over technology maturity alone. Terms like Ex‐Ante LCA and anticipatory LCA overlap with pLCA but highlight distinct nuances: Ex‐Ante LCA traditionally emphasizes technology upscaling to high technology readiness levels (TRL 9), though this requirement is not exclusive to pLCA; on the other hand anticipatory LCA often integrates stakeholder engagement to guide methodological choices, enhancing social relevance.^[^ ^7^ ^]^ Recent terminology reviews recommend unifying these under prospective LCA to reduce fragmentation.^[^ ^4^, ^7^ ^]^ Following this guidance, this work adopts pLCA as the primary term for all future‐oriented assessments.

Analyzing the pLCA definitions given by Arvidsson et al.,^[^ ^4^, ^7^ ^]^ the development of a pLCA involves three fundamental components: (1) Maturity level: defining the current stage of an emerging technology (e.g., experimental versus commercial); (2) Upscaling: modeling improvements in efficiency, material use, or production scale; (3) Future simulation: projecting supply chains, energy grids, and policy landscapes into the target timeframe.

A technology is considered “emerging” if it is in an early development phase and has yet to enter the market.^[^ ^5^ ^]^ This development phase is typically measured using technology readiness levels (TRL) or manufacturing readiness levels (MRL), which indicate the technology's progress beyond its initial stages.^[^ ^4^ ^]^ As TRL or MRL values increase, so does the understanding of the technology, suggesting reduced design flexibility and a shift toward growth and maturity phases.^[^ ^1^, ^4^ ^]^

The assessment of the initial stage of development of a technology is essential for ensuring comparability across data, system boundaries, and results.^[^ ^2^ ^]^ When a technology is at a low TRL, its functionality may not totally defined, potentially causing inconsistencies in the definition of system boundaries, co‐products and the functional unit.^[^ ^3^ ^]^ Furthermore, data availability and quality for low‐TRL technologies (e.g., lab‐scales, prototypes) are often limited, due to incomplete inventories and lack of historical data from well‐established industrial processes.^[^ ^2^, ^3^ ^]^

**Figure 1:**
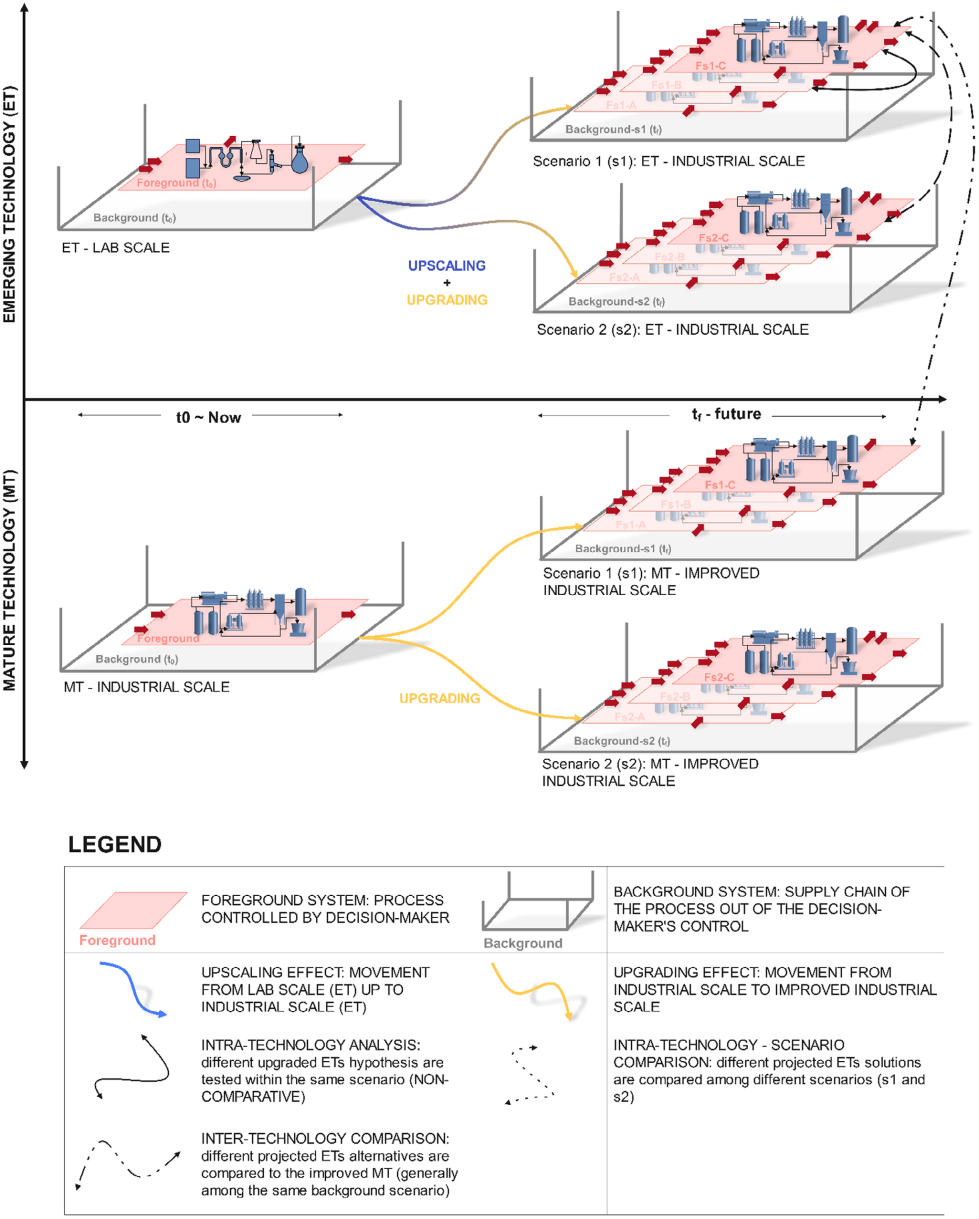
Graphical view of pLCA fundamental elements: MT is the Mature Technology available at time *t*_0_ (now); ET is the Emerging Technology at time *t*_0_,; MT‐IMPROVED INDUSTRIAL SCALE is the projection in the future time *t*_f_ of the Mature Technology MT, that improves due to technological learning and market diffusion according to two scenarios developed (s1 and s2); Fs1‐A, Fs1B, and Fs1‐C are possible upscaling simulations in the future time t_f_ of the initial ET within the s1 background scenario; at the same way, Fs2‐A, Fs2‐B, and Fs3‐C are possible scaled‐up solutions of ET within the s2 scenario.

For this reason, all technologies under assessment must be compared at similar maturity and complexity level, meaning that each technology must be scaled‐up to higher TRLs (e.g., industrial scale).^[^ ^3^ ^]^ According to Buyle et al. three factors must be considered to ensure the comparability: technological development, learning and diffusion.^[^ ^5^ ^]^ Technological development involves projecting the emerging technology's process from lower to higher TRLs. This upscaling approach is visually represented in Figure 1 by the “upscaling” concept (the blue‐based narrow), which project the lab‐scale ET to their more advanced (scaled‐up) alternatives, labeled as Fs1‐A, B, and C.

On the other hand, as a technology gains market presence, the knowledge base around it expands through the technological learning of the process and its market diffusion.^[^ ^5^ ^]^ In Figure 1, this concept is shown by a yellow‐based arrow (upgrading concept), indicating that both foreground and background systems of each emerging and mature technology need to be upgraded to simulate efficiencies, reference flows, and market conditions in hypothetical futures.

This approach addresses the need to prevent temporal mismatch between the foreground system – under the decision maker's control – and the background system, which encompasses the broader supply and may evolve independently over time.^[^ ^3^ ^]^ Such simulation of the future requires the application of scenarios.^[^ ^4^ ^]^

Developing alternative scenarios to contextualize scaled‐up technologies is valuable for multiple purposes. Firstly, it identifies environmental hotspots when evaluating the future‐oriented performance of emerging technologies for design purposes (e.g., in non‐comparative pLCA studies). Secondly, it enables intra‐technology comparisons by assessing projected alternatives of an ET within the same future scenario (e.g., s1), helping determine the most suitable scaled‐up version for specific applications. Thirdly, cross‐scenario comparisons of scaled‐up ET alternatives (intra‐technology scenario analysis) explore potential advancements independent of scaling, focusing instead on developments linked to background system enhancements (e.g., energy mix shifts or policy changes). Finally, comparing incumbent technologies with scaled‐up ETs within a defined timeframe (inter‐technology comparison) supports structured decision‐making by evaluating technologies at equivalent industrial maturity levels.^[^ ^2^, ^5^ ^]^ However, due to the numerous assumptions required, potential inconsistencies and lack of transparency, when generating future scenarios, are the main challenges to be managed during the development of pLCA.^[^ ^11^ ^]^

The following sections will detail these issues and address them using the latest methodologies from the literature, identifying the most critical aspects of pLCA and offering an overview of effective approaches for managing them.

## 3 Literature Data Analysis

To provide an overview of pLCA methodologies and their applications in case studies, a systematic literature review was performed to address two research questions:

Q1. What are the main methodological aspects of the pLCA and their critical aspects in implementation?
Q2. How are these methodological issues dealt with in recent published case studies?

Building on the work by Thonemann et al.,^[^ ^2^ ^]^ the conducted literature review follows a four‐phases structure:^[^ ^12^ ^]^ source identification, source selection, source evaluation, data analysis. The identification phase took place from July to August 2024, using the peer‐reviewed academic databases of Web of Science and Scopus. Detailed information about each review phase can be found in Supporting Information.

The resulted lists of 79 articles were grouped into three categories: Literature Review, Case Studies, and Methodology/Other. The Literature Review category includes articles reviewing the full scope of pLCA development or focusing on specific components, such as upscaling methods. The case studies includes studies explicitly identified by their authors as pLCA. The Methodology/Other category encompasses articles relevant to pLCA development that do not fit into the previous categories, such as methodological papers. Articles classified in this latter category are further divided into sub‐categories as reported in Table 1.

**Table 1:** Final number of articles related to the pLCA development and the research questions.

Category^]^	Sub‐category	Number of articles
Literature review	‐	12
Methodology/other	Guide/method for whole pLCA	1
	Guide/method for part pLCA	16
	Application of a methodology in case study	10
	External/linked observation:	11
Case studies		29
Total	‐	79

The analysis of the classified articles was conducted in two phases. The first phase aimed to answer Q1 by identifying the most relevant methodological procedures from the articles categorized as Literature Review and Methodology/Other that address key pLCA challenges. In the second phase these identified methodologies were used as a benchmark to examine the case studies (addressing Q2). For more information, please refer to Supporting Information.

## 4 Critical Aspects and Methodological Procedures

**Figure 2:**
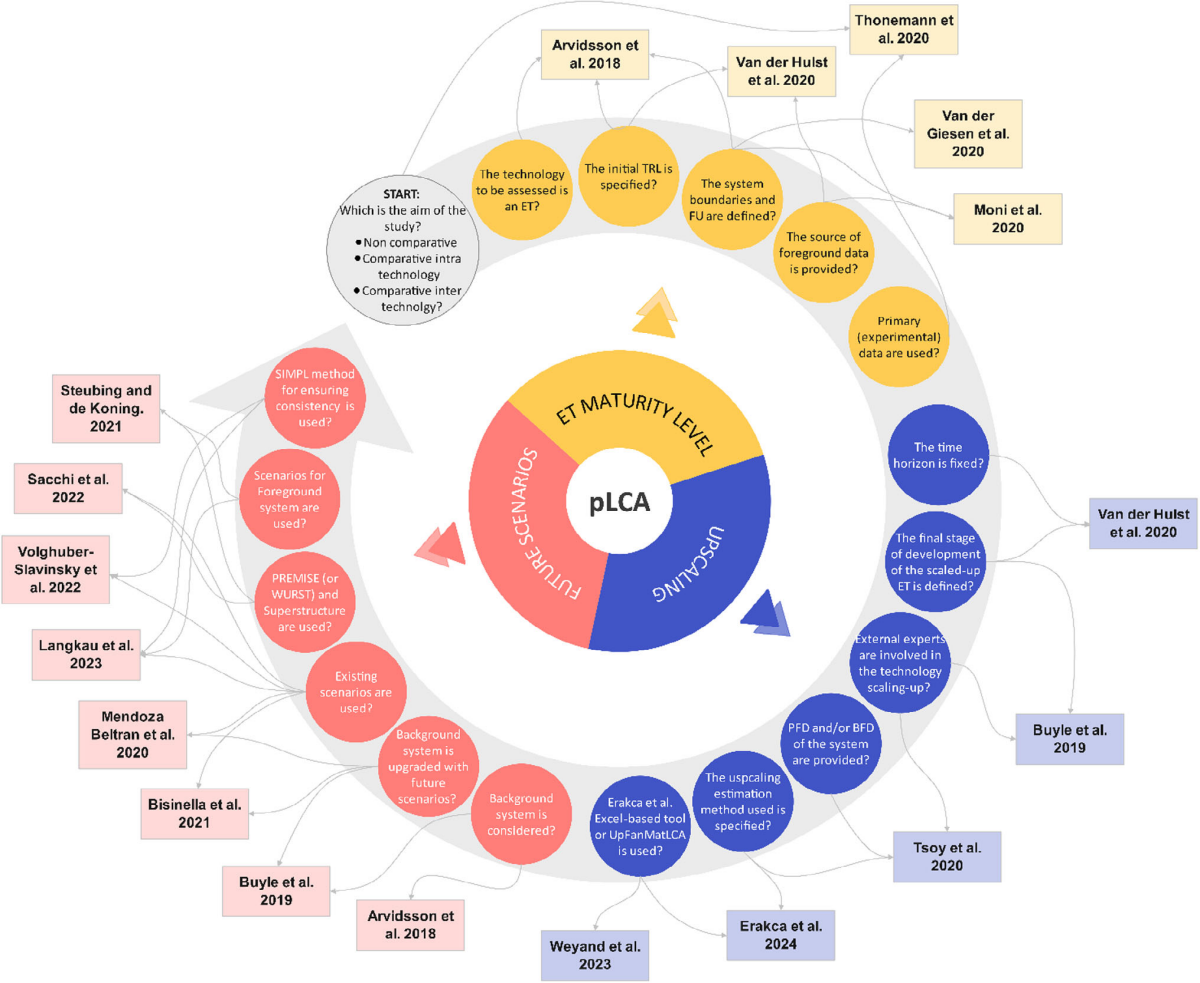
Main aspects extracted from literature analysis and required for the development of a robust pLCA; these methodological aspects are then used as benchmark for the analysis of case studies. Each aspect is associated to the articles where it comes form and the relative area, derived from the pLCA definition: ET maturity level, upscaling, and future scenarios. Subsequently, these identified criteria are contextualized within the traditional LCA phases to produce a step‐by‐step guide to be used as updated framework for developing robust pLCA.

The first phase involved analyzing articles categorized as Literature Review and Methodology/Other. The literature reviews served to identify key challenges, while methodological papers were used to evaluate possible solutions to address these criticalities. Consequently, drawing on insights from these studies, the main aspects for developing a robust pLCA are extracted and outlined (Figure 2).

### 4.1 Emerging Technologies Maturity Level

Conducting a prospective life cycle assessment (pLCA) poses heightened challenges compared to traditional LCAs.

The environmental impacts assessed for technologies at low technology readiness levels (TRLs) may not accurately represent their final state due to various limitations associated with early‐stage development. These include restricted access to primary data, challenges in defining comprehensive functionalities and system boundaries, and difficulties in selecting appropriate impact categories.^[^ ^1^, ^2^, ^13^ ^]^

Thonemann et al. emphasize that data quality and availability are strongly influenced by a technology's initial TRL.^[^ ^2^ ^]^ Furthermore, low TRLs hinder study comparability, as functionalities and system boundaries may remain ambiguously defined at early development stages.^[^ ^2^, ^13^ ^]^ In other terms, the low maturity degree of ETs complicates the precise definition of FU, limiting the adequate selection of the technologies alternatives to be compared, and further leading to inconsistencies in modeling choices.^[^ ^4^ ^]^ While Moni et al. acknowledge similar issues, they address scaling as a standalone concern,^[^ ^3^ ^]^ whereas Thonemann et al. frame it within broader data‐related challenges.^[^ ^2^ ^]^

Regarding impact assessment, evaluating ETs requires careful consideration of limitations inherent to life cycle impact assessment (LCIA) methodologies. As noted by Thonemann et al. and Van der Giesen et al.,^[^ ^2^, ^13^ ^]^ LCIA limitations may compromise the holistic evaluation of environmental burdens. ETs’ characteristics are often poorly understood, leading to undefined relevant impact categories, imprecise burden allocations, and potentially flawed decision‐making.

These factors collectively amplify uncertainties in pLCA. Parameter uncertainty—driven by unreliable input data—is exacerbated in early‐stage technologies due to inconsistent data sources. Additionally, complexities in LCIA selection, FU definition, and system boundary delineation increase scenario and model uncertainties compared to conventional LCA. This arises from the assumptions necessitated during the Goal and Scope phase.^[^ ^2^ ^]^

To address some of these challenges, Van der Hulst et al. propose a systematic approach for ET environmental evaluation.^[^ ^1^ ^]^ Their method comprises three phases: the definition stage, where the technology's TRL is specified to determine which steps are required for suitable results; the process changes phase, in which size scaling and the optimization of foreground flows are carried out; and a final phase where background data are upgraded and combined with scenarios. The key point proposed by Van der Hulst et al. is the importance of defining the technology's initial development stage to gauge the confidence's degree of data, identify the source of foreground system data, and select the appropriate scaling‐up for projecting LCI data to higher TRLs.^[^ ^1^ ^]^ Once the TRL of a technology has been determined, Arvidsson et al. propose two distinct approaches for the prospective selection of alternative technologies to be compared. The first approach focuses on a specific functionality (FU), analyzing from a forward‐looking perspective which technologies currently classified as ET could potentially fulfill that function in the future. The second approach, instead, centers on each individual ET process through a cradle‐to‐gate analysis, assessing all possible applications and functionalities, thereby shifting the perspective of evaluation.^[^ ^4^ ^]^

The several potential origins of foreground systems’ data were listed by Van der Giesen et al., including unpublished lab experiments, pilot projects and process simulations, scientific articles, expert interviews, and patents.^[^ ^13^ ^]^ Specifically for patent data, Spreafico et al. develop a systematic approach that integrates pLCA with patent analysis in order to support the LCI system modeling and to extract technical information that meets the data quality standards.^[^ ^14^, ^15^ ^]^ Moreover, Thonemann et al. specify also that primary data should be preferred to increase the LCI quality.^[^ ^2^ ^]^ An alternative route, as described by Salla et al.^[^ ^16^ ^]^ in their review, might be represented by the application of machine learning (ML) methods, such as artificial neural networks (ANNs) and the adaptive neuro‐fuzzy inference system (ANFIS) to manage imprecise and uncertain data, establishing relationship between variables and enhancing the model robustness by reducing overfitting on data.^[^ ^16^ ^]^

### 4.2 Upscaling Methods

After defining the study's goal and selecting ET alternatives, the collected data for the foreground flows must be modeled at the future time $t_{f}$, when the ET production scale is expected to be comparable to the mature technology.^[^ ^4^ ^]^ Therefore, the collected data from the foreground system must be scaled up to a more advanced stage with a higher TRL while ensuring alignment with the fixed time horizon. This projection introduces additional uncertainties into the model, as it involves not only incorporating more uncertain parameters due to the low TRLs of ET but also forecasting these data into a future, more developed stage, adding an extra layer of scaling complexity.^[^ ^2^, ^3^ ^]^

To manage these problems, Buyle et al. propose a significative framework outlining possible techniques for scaling up ETs and achieving efficiency and productivity gains through learning‐by‐doing and market diffusion, also referred to as the upgrading step.^[^ ^5^ ^]^ Moreover, they stressed out the need to differentiate between methods for modeling the foreground system and techniques for upgrading the background system due to the varying levels of detail required.

Similarly, Van der Hulst et al. claim that changes in background process should be viewed as external developments outside the control of decision‐maker, but able to influence the technological advancements in the foreground both upstream and downstream.^[^ ^1^ ^]^ Therefore, the authors suggest that background inventory data should align temporally with the projected foreground LCI data, which in turn need to be scaled up with respect to their low TRL‐acquisition status in order to allow the suitable comparison of results.

Tsoy et al. also categorized data estimation methods used in literature, including process simulation, manual calculations, molecular structure models (MSMs), finally developing a decision tree o guide the selection of appropriate estimation methods.^[^ ^17^ ^]^

A step forward was done by Erakca et al., which introduce new data estimation methods and an innovative excel‐based tool that improves upon the decision tree proposed by Tsoy et al.^[^ ^6^, ^17^ ^]^ According to their review, the main estimation methods for constructing the LCI foreground‐dataset include approximation, process engineering, simple extrapolation, simulations, advanced empirical scaling, modular influence estimation and MSMs.

A notable example of process engineering can be seen in Elginoz et al. (2022), where an engineering‐based upscaling method was used to generate LCI data for biochemical resource recovery processes.^[^ ^18^ ^]^ Simulations methods are illustrated by Kikuchi and Kanematsu and Kikuchi et al., who compensate the intrinsic data limitation in pLCA studies by utilizing Computer‐aided process engineering (CAPE) tools, validated through case studies on acetylated cellulose nanofiber‐reinforces plastics and recycling system design of Lithium‐ion batteries, respectively.^[^ ^19^, ^20^ ^]^

Faber et al. describe instead a methodology for incorporating technology learning curves (TLCs) into LCA phases to anticipate the economic and environmental performances of emerging carbon capture and utilization (CCU) technologies.^[^ ^21^ ^]^ For further examples of these upscaling estimation methods in practice, refer to the work by Erakca et al.^[^ ^6^ ^]^

### 4.3 Upgrading Approaches and Future Scenarios

Future scenarios are required to address the need to avoid temporal mismatches between foreground and background data,^[^ ^1^, ^4^ ^]^ as well as to contextualize the scaled‐up ET within a range of plausible and realistic futures.^[^ ^5^, ^11^, ^22^ ^]^ However, this contextualization required additional assumptions about future developments introducing additional epistemic uncertainties linked with modeling choices and future scenario developments.^[^ ^8^ ^]^

Bisinella et al. provide a comprehensive review of future scenarios and their application in LCA, emphasizing that these scenarios has not to be interpreted as predictions, but rather as a plausible alternative visions of the future.^[^ ^8^ ^]^ They also outline the stage of scenario building and highlight the importance of choosing among predictive, explorative and normative scenario types. However, Bisinella et al. emphasizes the importance of scenario analysis as a tool for systematically addressing epistemic uncertainty in future background scenario modeling, highlighting that, the incorporation of scenario analysis alongside uncertainty and sensitivity analyses is a key approach to effectively managing uncertainties.^[^ ^8^ ^]^ Additionally, the same authors critique that although some articles project data into future, they fail to observe the SETAC guidelines,^[^ ^23^ ^]^ for future scenarios, undermining the communicability and representativeness of pLCA results.^[^ ^8^ ^]^

To fulfill these issues, Langkau et al. propose the SIMPL method, a stepwise approach for scenario‐based inventory modeling, which integrates into the first two LCA phases required assumptions to build scientifically rigorous future scenarios.^[^ ^22^ ^]^ This approach complements the technological upscaling methods proposed by Erakca et al. and Tsoy et al., covering the entire process of prospective LCI data modeling and adding the possible alternative layers for both foreground and background data originated from different scenarios application.^[^ ^6^, ^17^, ^22^ ^]^ Langkau et al. also emphasize a hierarchy of assumption sources, recommending that existing scenarios should be the primary source for assumptions, particularly for background data due to complexity of simulating sector interconnections.^[^ ^22^ ^]^ When existing‐quantitative scenarios are not available, assumptions can be (i) developed using literature and expert knowledge, creating tailored scenario primarily for foreground LCI data modeling, or (ii) derived from qualitative scenarios that typically outline the development of specific background systems.^[^ ^22^ ^]^ In this context, Volghuber‐Slavinsky et al. made significant contributions by proposing a methodology to convert qualitative scenarios into quantitative data, with a focus on the agri‐food sector.^[^ ^24^ ^]^

A notable advancement in integrating existing scenarios with pLCA development is offered by Mendoza Beltran et al., who suggest using integrated assessment models (IAMs) together with the database Ecoinvent.^[^ ^11^, ^25^ ^]^ IAMs are models that translate the socioeconomic conditions from the shared‐socioeconomic‐pathways (SSPs) into projections among different years of future energy use characteristics and GHGs emissions. Building on this work, Sacchi et al. developed PREMISE, a tool that enhances IAMs integration into Ecoinvent, allowing for the creation of projected background pLCI databases across various future timelines aligned with specific IAMs and SSP scenarios.^[^ ^26^ ^]^

In addition to PREMISE, Steubing and de Koning introduced the Superstructure approach, a comprehensive LCI database structure capable of managing numerous pLCI databases across different scenarios, thereby simplifying the management and ensuring compatibility across various scenario‐based databases.^[^ ^27^ ^]^

A noteworthy contribution comes from Weyand et al. who introduced an innovative systematic framework, called UpFunMatLCA, for generating exploratory scenarios tailored to emerging functional materials (FunMat)‐based energy technologies.^[^ ^28^ ^]^ This scheme facilitates the structured creation of both qualitative and quantitative scenarios, which can be integrated into the LCI phase to realistically depict potential development pathways for FunMats. By following the three steps of UpFanMatLCA, named as (i) upscaling definition, (ii) upscaling leap, and (iii) upscaling model and data, LCA practitioners are guided in selecting pathways that represent possible future design choices.

## 5 Case Studies Assessment

The second phase of the analysis focused on addressing Q2 by using the identified methodologies as benchmark to evaluate the degree of completeness of 20 pLCA case studies published after 2021. The results are summarized in Table 2, where case studies are listed at the top, and key aspects derived from Q1's answers are grouped according to their area of definition as already depicted in Figure 2. Therefore, when the case study in analysis uses or incorporates in its development the identified methodology, a positive mark (P) is assigned; vice versa, if the indicated methodology is absent, a negative mark (O) is used. A distinct approach was applied to the initial aspect, classifying studies based on their aim into non‐comparative (NC), comparative inter‐emerging technologies (CIetT), comparative inter‐technologies (CIT), as already schematized in Figure 1.

**Table 2:** Prospective LCA case studies from 2021 to 2024 analyzed in order to provide an answer to Q2: the analysis has been conducted using as benchmark the main aspects identified in Q1. Articles not cited elsewhere in the text.^[^ ^29^, ^30^, ^31^, ^32^, ^33^, ^34^, ^35^, ^36^, ^37^, ^38^, ^39^ ^]^

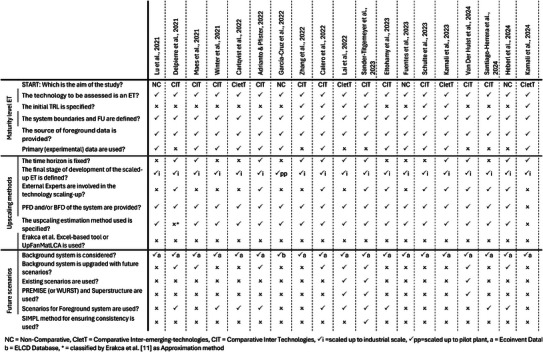

Under the ET maturity level area, all studies treat Emerging Technologies, defining functional units, system boundaries, and reporting foreground LCIs, which are largely based on primary or mixed data sources. More critical is the TRL specification; in fact, only 7 studies explicitly stated initial TRLs of the analyzed ET, predominantly between 4 and 7, while the remaining case studies provide only a general description of the initial technology readiness level. The absence of a clear TRL specification provided by the authors can limit the result comparability and the ability to select appropriate scaling‐up methodologies. ^[^ ^4^, ^6^, ^28^ ^]^

Greater homogeneity seems to be present in the upscaling area, as all case studies address the upscaling of ET. Industrial scale is the predominant upscaling level, applied in 19 articles out of 20. Only Garcia‐Cruz et al. developed a pLCA on a based orange wax fungicide, scaling‐up data from lab‐scale to pilot‐plant.^[^ ^40^ ^]^ However, even if all the studies develop the LCIA phase based on scaled‐up data, comparability is hindered by the absence of a fixed time horizon and the diversified use of scaling methodologies without standardized frameworks, such as UpFanMatLCA or the Erakca et al.'s Excel‐based tool.^[^ ^6^, ^28^ ^]^

Starting from this latter issue, the diversity in projecting data to higher TRLs depends on the aim of the study, data quality, and the LCA practitioners' expertise.^[^ ^6^, ^17^ ^]^ For this reason, the involvement of ET experts is carefully recommended but only the 55% of the papers specifically express the integration of external experts in the pLCA development. Adrianto and Pfister exemplify effective integration, scaling‐up in‐house experimental data of sulfidic copper tailings via expert judgement, engineering‐based calculations, and proxies.^[^ ^41^ ^]^

Another notable work, published by Schulte et al., analyzed a case study on waste electrical and electronic equipment recycling by pyrolysis, introducing also a pLCA framework for emerging recycling technologies.^[^ ^42^ ^]^ They addressed the intrinsic multifunctionality issue in end‐of‐Life processes, developing a series of steps to tackle the challenges in foreground LCI data collection for recycling activities. However, their focus remained on the foreground system, omitting clear timeframes and background system upgrades since, according to the authors, legislation can directly affect the future scenarios.

Generally, the lack of a fixed time horizon can reduce the congruency of studies, as scaled‐up technologies at higher TRLs might not align with the indicated time frame, because different technologies may require varying development periods to reach maturity. Furthermore, the absence of a specific time horizon inhibits accurate upgrades to the background system, causing the mismatch between foreground and background LCI data. Notably, nine studies show a background system upgrade, and all of them also include a clearly defined time horizon.

Calero et al. provided a significative application of these concepts, conducting an in‐depth study of a pasteurization technology, starting at TRL 7 and advancing to TRL 9 over a 20‐year timeframe.^[^ ^43^ ^]^ This progression involved contributions from technology experts and industrial stakeholders. They also developed multiple scenarios, mainly focusing on the foreground system while accounting for changes in the background system, such as a revised electricity market mix and methane emission reductions in Spain by 2040.

Conversely, some studies intentionally omitted background system upgrades due to shorter time horizons. Zhang et al. proposed a study on flexible all‐organic battery, scaling‐up the system from a laboratory‐scale (TRL 4) to industrial one, fixing a time horizon of 10 years.^[^ ^44^ ^]^ The short‐term time horizon, has prompted the authors to consider the current background situation, building on suggestions put forward by Arvidsson et al.^[^ ^4^ ^]^ Similar conclusions were also achieved by Kamali et al. in a pLCA on sustainable electrochromic displays, where given the use of a 5‐year time horizon, made it appropriate to select the current electricity mix.^[^ ^45^ ^]^

Focusing instead on the future scenario area, the most relevant source of inconsistency is the limited adoption of already existing scenarios. Among the nine studies that upgrade the background system, only three articles utilized well‐developed existing scenarios. This gap is evident when comparing the above‐cited study by Calero et al. with Van der Hulst et al.’s work on Silicon and Silicon/Perovskite Tandem Photovoltaics.^[^ ^43^, ^46^ ^]^ The latter utilized the IMAGE IAMs with the specific SSP2 projection as existing through the PREMISE tool,^[^ ^26^ ^]^ which not only adjusts the electricity market mix of background system over time, as done by Calero et al.,^[^ ^43^ ^]^ but also incorporates the learning‐by‐doing effects and efficiency improvements across several industrial sectors,^[^ ^26^ ^]^ ensuring consistent and future‐oriented upgrades of background data.

With a different purpose, but applying similar methodologies for future scenarios, Lai et al. explored the large‐scale application of sustainable aviation Fuel in Sweden through a socio‐technical approach to pLCA, providing results specifically designed to be used in policies and investment decisions.^[^ ^47^ ^]^ To upgrade the background system, they employed the Superstructure approach and Wurst software^[^ ^11^ ^]^ to incorporate future scenarios in the electricity sector, including Sweden's projected 2030 electricity mix.^[^ ^11^, ^27^ ^]^

Therefore, in order to enhance the consistency and comparability of results, the utilization of existing scenarios must be preferred to the production of ad‐hoc future scenarios.^[^ ^22^ ^]^

In this context, Sander‐Titgemeyer et al., utilize the SIMPL approach in a pLCA on three emerging wood‐based technologies: Lignin‐based adhesive – LPF (TRL 4), glued‐laminated (Glulam) lead‐bearing beam (TRL 7), and cellulose‐based viscose fiber for textiles (TRL 9).^[^ ^48^ ^]^ They generated LCI data by involving technology experts, using upscaled data primarily derived from simulation software, and modeling the background system through existing scenarios, based on Mendoza Beltran et al.’s concepts.^[^ ^11^ ^]^ The application of the SIMPL methodology resulted in the individuation in the Bioeconomy Strategy as external factor capable of influencing the foreground system, producing five consistent scenarios for LPF case, two for Glulam beam, and three for viscose in textiles. The future scenarios selected by the authors consider different combinations of status‐quo conditions, improvements provided with the application of renewable energy and different assumptions in the foreground system upscaling.

### 5.1 Dealing with Uncertainty

Given the numerous sources of uncertainty in pLCA studies, which amplify uncertainty levels compared to traditional LCA, a systematic uncertainty analysis is crucial for accurately interpreting results.^[^ ^48^ ^]^ However, the approaches used for result analysis in the reviewed case studies are far from uniform.

Most studies employ contribution and hotspot analyses to identify primary impact drivers across alternatives. Yet, due to the forward‐looking nature of pLCA, integrating scenario analysis is essential to address uncertainties stemming from modeling assumptions (e.g., technological adoption rates or policy shifts).^[^ ^8^, ^22^ ^]^ Despite this, only 9 of the 20 analyzed studies explicitly incorporate scenario analysis.

Data uncertainties, meanwhile, are often quantified through sensitivity and uncertainty analyses. Sensitivity analysis—adjusting input parameters to assess outcome variability—is applied in 13 studies. By contrast, uncertainty analysis (e.g., Monte Carlo simulations), which accounts for inherent data variability, is implemented in only 5 studies.

A noteworthy analysis of results can be found in Fuentes et al.,^[^ ^29^ ^]^ which evaluates the environmental impacts of magnetite nanoparticle (MNP) synthesis in microfluidic devices. The study begins with a contribution analysis, identifying key hotspots at both laboratory and industrial scales. It carefully examines the influence of electricity consumption, transportation, and chemical use, as well as the impacts associated with the manufacturing process, operational phase, and wastewater management. Following this, a scenario comparison is conducted through a sensitivity analysis, exploring two alternative materials as substitutes for rivets—identified as a critical factor influencing microfluidic device manufacturing based on the contribution analysis. Additionally, a second sensitivity analysis is performed, focusing on background parameters, particularly comparing the effects of different electricity mixes. Finally, a Monte Carlo simulation with 1000 iterations and a 95% confidence interval is carried out to assess the reliability of the results.

Another significant example is Heberl et al.,^[^ ^30^ ^]^ which investigates biological methanation in a trickle‐bed pilot plant across different scales and future scenarios. The study analyzes the process at its current scale, a scaled‐up version for 2024, and two additional scaled‐up versions contextualized within 2050 best‐ and worst‐case scenarios. A hotspot analysis across all scenarios highlights the impact of upscaling data, revealing a shift in key contributors: while electricity for methanation is the dominant factor at the current scale, in the three scaled‐up scenarios, the main contributor shifts to operations for electrolysis. Additionally, the worst‐ and best‐case scenarios undergo a Monte Carlo simulation with 10,000 iterations to identify the most probable outcomes.

A special mention goes to Sander‐Titgemeyer et al.,^[^ ^48^ ^]^ which not only incorporates uncertainty and sensitivity analyses but also introduces a structured uncertainty management approach. In this method, uncertain parameters are classified as scenario‐related or scenario‐independent, with a particular focus on the first category to enhance the development of future scenarios.

### 5.2 Key Lessons Learned

The analysis of recent case studies revealed both notable contributions and limitations in the application of pLCA methodologies. While all studies addressed the upscaling of emerging technologies to higher TRLs, the diversity in upscaling methods and the absence of a fixed time horizon hindered the uniformity of results. Furthermore, the limited adoption of existing scenarios for upgrading background systems introduced inconsistencies and potential biases. This heterogeneity reduces the effectiveness and comparability among the pLCA studies, causing inconsistencies and uncertainties which limit the adoption of the pLCA methodology. Therefore, a more comprehensive framework to guide the pLCA development has been produced to partially reduce the variability of approaches adoptable, guiding the choice among the available techniques to find the most suitable methodologies specifically shaped on the conditions of each single analysis. This guideline provides a structured approach to selecting appropriate upscaling techniques based on the initial TRL and data availability, while also promoting the utilization of well‐established scenarios for consistent background system upgrades.

**Figure 3:**
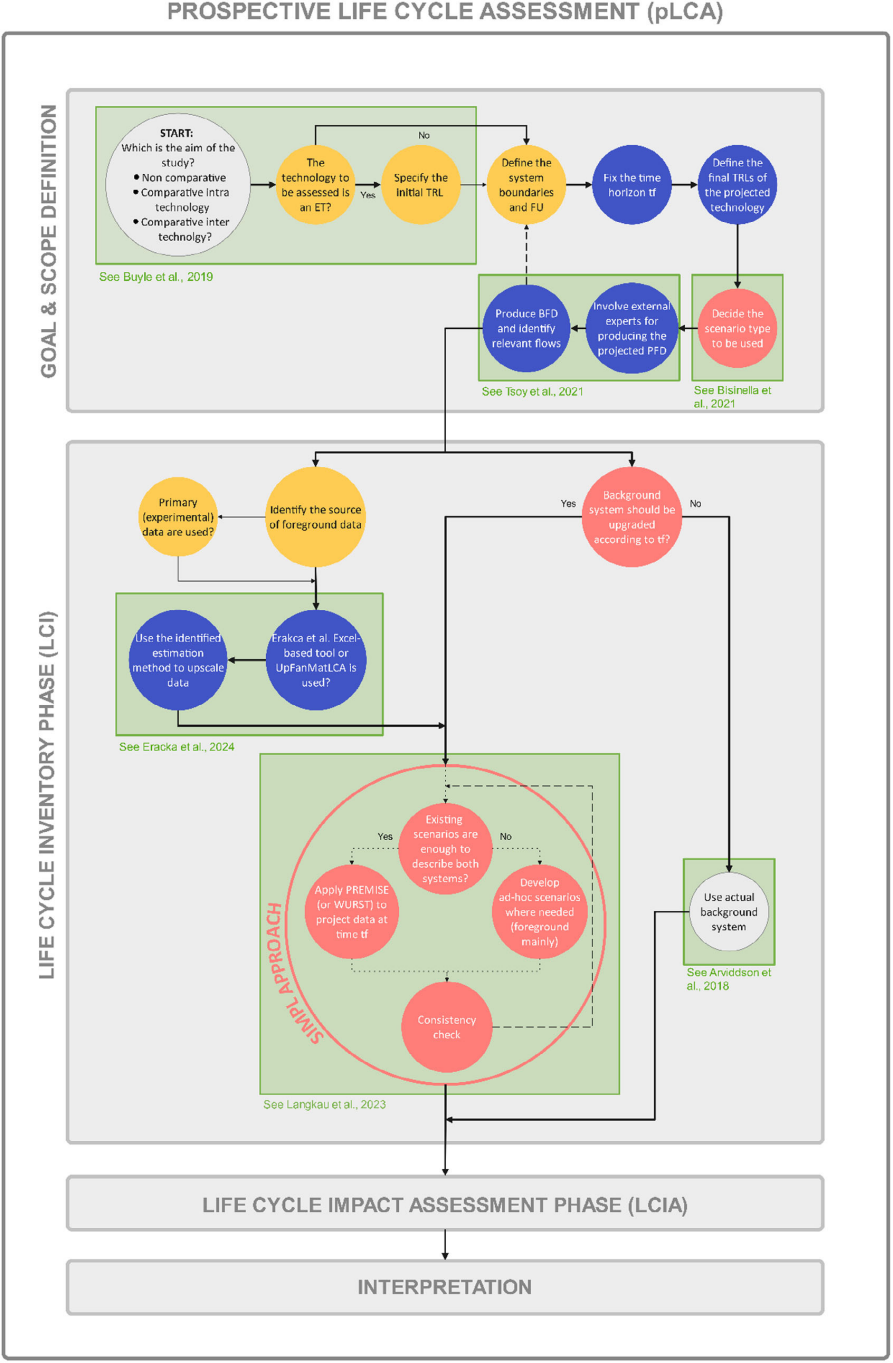
Step‐by‐step guide for pLCA study development: the identified methodological criteria are contextualized within the traditional LCA phases to obtain a structured updated framework to guide the LCA practitioners in the choice of the suitable techniques relying on the most recent advancements in the field.

In Figure 3, a simplified flowchart provides a guide for integrating pLCA development into the traditional LCA framework. This scheme incorporates the methodological criteria outlined in Figure 2, contextualizing them within each of the four LCA development phases. By synthetizing information from the literature review and case study analysis, the flowchart presents a rigorous up‐to‐date approach based on the most relevant methodologies and techniques, ensuring a systematic application of pLCA principles.

This flowchart relies on a logical progression from the Goal and Scope phase to the LCI phase, integrating key criteria essential for scope definition and data management. It influences system boundary delineation, functional unit selection, and reference flows, as well as pointing out recent advancements in upscaling techniques and scenario development, hence assisting users in selecting the most appropriate methodology based on a structured approach rather than a holistic one.

Following this step‐by‐step guide, the robustness and comparability of pLCA studies is enhanced, reducing the degree of uncertainties linked with methodological choice and facilitating a more informed decision‐making for the sustainable development and implementation of emerging technologies.

However, the management of epistemic and data uncertainties remains a critical challenge that requires further attention. While the reviewed methodologies, as contextualized within the guideline framework, contribute to reducing variability and inconsistencies in pLCA modeling choices, a more systematic approach is needed to standardize the quantification and management of these uncertainties while ensuring effective communication.

A potential solution, proposed by Heijungs,^[^ ^49^ ^]^ is to clearly distinguish between sensitivity and uncertainty analyses rather than treating them as a dual‐tandem approach. By using the results of uncertainty analysis on data to inform subsequent sensitivity analysis, it becomes possible to assess how variations in already‐tested data and methodological choices impact the results.^[^ ^49^ ^]^ This would improve transparency and reliability, allowing stakeholders to make well‐informed decisions based on a clearer understanding of the underlying assumptions and limitations. In the same context, the integration of Machine Learning and AI‐driven tools with pLCA methodologies could offer an innovative means to strengthen model robustness, particularly in data regression and classification tasks.^[^ ^16^ ^]^ Specifically, applying sequential decision algorithms to determine the most appropriate upscaling methodology based on the characteristics of collected data could help mitigate the pLCA methodological heterogeneity. However, challenges related to data quality and availability persist also in this field, limiting the effectiveness of machine learning approaches.^[^ ^16^ ^]^

Overall, this analysis underscores the importance of ongoing research efforts to refine and standardize pLCA methodologies, ensuring their effective application in evaluating the environmental implications of emerging technologies. By proactively capturing future technological developments and their relevance to specific market scenarios, pLCA can serve as a valuable predictive tool for decision‐makers. From a private sector perspective, it can support strategic business improvements, while in the public sector, it can inform the development of policy roadmaps. Additionally, pLCA could become a key technique in the technology design stage, allowing for the early identification of potential challenges during the development phase, when technological flexibility still permits design modifications. In this way, the effective application of pLCA methodologies can anticipate technological implications with a forward‐looking perspective, contributing to the transition toward a more sustainable future.

## 6 Summary and Outlook

This study provides a comprehensive review of pLCA, an emerging methodology for evaluating the environmental impacts of technologies still in the development phase. The adoption and study of pLCA are gaining significant momentum, driven by the methodology's unique focus on proactively shaping sustainable futures rather than retrospectively evaluating past impacts. Through a systematic literature analysis, critical aspects of pLCA were identified, including assessing technology maturity levels, upscaling methods to model future industrial scales, and generating future scenarios. Recent case studies were analyzed to examine the implementation of these methodological aspects, revealing notable contributions as well as limitations that require further attention.

The findings underscore the importance of pLCA as a valuable tool for researchers and scientists engaged in the development of advanced technological and material innovations. By integrating pLCA early in the design process, potential environmental hotspots can be identified and mitigated before technologies reach maturity and widespread adoption, when design modifications become increasingly challenging. This proactive approach aligns with the principles of eco‐design and supports the transition towards a more sustainable future.

However, the analysis also highlighted the need for more comprehensive guidelines that integrate scenario development with upscaling methods. For this reason, a synthetic step‐by‐step approach has been proposed to guide in the methodological choices during pLCA development, enhancing the robustness and comparability of pLCA studies, facilitating informed decision‐making for the sustainable development and implementation of emerging technologies. However, a more systematic approach to quantifying and communicating uncertainties associated with pLCA studies is necessary to improve transparency and reliability.

Looking ahead, continued research efforts are crucial to refine and standardize pLCA methodologies, ensuring their effective application in evaluating the environmental implications of emerging technologies. Collaboration between LCA practitioners, technology experts, and stakeholders is essential to address the remaining challenges and promote the widespread adoption of pLCA as a valuable tool for sustainable innovation.

As researchers and scientists continue to push the boundaries of technological advancements, the integration of pLCA into the development process becomes increasingly vital. By anticipating and mitigating potential environmental impacts, pLCA can guide the design and implementation of emerging technologies, ensuring their contribution to a more sustainable future while supporting the achievement of global sustainability goals.

## Supporting Information

The authors have cited additional references within the Supporting Information.^[^ ^50^, ^51^, ^52^, ^53^, ^54^, ^55^, ^56^, ^57^, ^58^, ^59^, ^60^, ^61^, ^62^, ^63^, ^64^, ^65^, ^66^, ^67^, ^68^, ^69^, ^70^, ^71^, ^72^, ^73^, ^74^, ^75^, ^76^, ^77^, ^78^, ^79^, ^80^, ^81^, ^82^, ^83^, ^84^, ^85^, ^86^, ^87^ ^]^

## Conflicts of Interest

The author declare no conflicts of interest.

## Supporting information



Supporting Information

## Data Availability

The data that support the findings of this study are available in the supplementary material of this article.
