# Supplementary material for: Looking to the Future: Prospective Life Cycle Assessment of Emerging Technologies
Source: Chemistry. 2025 Apr 3;31(25):e202500304. doi: 10.1002/chem.202500304 (PMC12057593; doi:10.1002/chem.202500304)
Supplement: Supplementary file 1 — Supporting Information [file CHEM-31-e202500304-s001.docx]

Chemistry – A European Journal

Supporting Information

**Looking to the future: prospective Life Cycle Assessment of emerging technologies**

Alessandro Marson,* Alberto Benozzi, and Alessandro Manzardo

# **Supporting information**

Looking to the future: prospective Life Cycle Assessment of emerging technologies

Alessandro Marson,*^[a]^ Alberto Benozzi,^[a]^ and Alessandro Manzardo^[a]^

[a] Dr. A. Marson, Mr. A. Benozzi, Prof. A. Manzardo
CESQA (Quality and Environmental Research Centre) - Department of Civil, Environmental and Architectural Engineering, 
University of Padova
Via Marzolo 9, 35131, Padova, Italy
E-mail: alessandro.marson@unipd.it

Supporting information for this article is given via a link at the end of the document.

## Review methods

The identification phase took place from July to August 2024, using the peer-reviewed academic databases of Web of Science and Scopus. The search strings chosen were those defined by Thonemann et al. in their review ^[2]^: ("emerging technolog*" OR "novel product*" OR "technolog* maturity" OR "early research technolog*" OR "scaling technolog*" OR "laboratory plant*" OR "pilot plant*" OR "novel process*" OR "early research") AND ("life cycle assessment" OR "prospect* LCA" OR "scal* LCA" OR "ex-ante LCA" OR "project* LCA" OR "predict* LCA" OR premise" OR "integrated assessment mod*" OR "LCI*"). The last three strings were added to make the extraction more selective, including the latest literature developments.

The resulted lists of articles were further filtered by considering only those in English, published after 2019 (included) in their final version, and excluding the unrelated areas of Medicine, Art and Humanities, Pharmacology, Toxicology and Pharmaceutics, Immunology and Microbiology, Health Professions from Scopus’ extraction and areas of Health care sciences services, medical informatics, microbiology, oncology, meteorological atmospheric sciences from Web of Science one. Finally, using an Excel-based tool DOI code checker, the two lists were merged, removing duplicates. During the Source evaluation phase, two additional articles, not part of the initial extraction, were included, bringing the total to 667 articles.

According to their relevance, the articles were categorized into three levels of usefulness:

1. Useful (U): articles with titles containing the terms "Prospective", "Ex-Ante", or "Ex Ante".
2. To Be Checked (TBC): articles with titles containing terms like “Predictive”, “Upscaling”, “Future-Oriented”. A further manual check was carried out based on the title analysis. If the title suggested a possible connection to pLCA, the article was inserted in the TBC level.
3. Not Useful (NU): articles considered not relevant after the manual check for the pLCA review.

After that, articles in the TBC level were further evaluated through a dedicated abstract analysis, distinguishing articles in useful (TBC-U) and not useful (TBC-NU). The final list of useful articles is made by 79 elements, as shown in Table S1.

**Table S1**: Results from literature analysis conducted between July-August 2024, considering only articles published between 2019 to 2024; in the first section the number of elements of the two extraction are reported together with the articles part of the complete merged list, the second one contains the usefulness level subdivision among Useful (U), To be checked (TBC) – further divided into Useful TBC (TBC-U) and not Useful TBC (TBC-NU) – and Not useful (NU). Finally, the last section reports the classification of the 79 kept articles, which are grouped in Literature review, Methodology/other and Case studies

| Source Identification: Number of articles | Extraction from Scopus | | Extraction from Web of Science | Merged list (no duplicates) | | | |
| --- | --- | --- | --- | --- | --- | --- | --- |
|  | 495 | | 508 | 667 | | | |
| Source Selection: usefulness analysis |  | | | U | TBC | | NU |
|  |  |  |  | 48 | 138 | | 481 |
|  |  |  |  |  | TBC-U | TBC-NU |  |
|  |  |  |  |  | 31 | 107 |  |
| Final number of articles for the Source Evaluation |  | | | Kept | | Rejected | |
|  |  |  |  | 79 | | 588 | |
|  | Literature review | | | 12 | |  | |
|  | Methodology/other | | | 38 | |  |  |
|  |  | *Guide/method for whole pLCA* | | *1* | |  |  |
|  |  | *Guide/method for part pLCA* | | *16* | |  |  |
|  |  | *Application of a methodology in case study* | | *10* | |  |  |
|  |  | *External/linked observation:* | | *11* | |  |  |
|  | Case studies | | | 29 | |  |  |

In order to address the key questions Q1 and Q2 of the review, the 79 final articles were grouped into three categories: Literature review, Case studies and Methodology/other. The first two categories contain the literature reviews on pLCA or its components (e.g. upscaling methods), and case studies defined by the authors as pLCA according to the definition of Arvidsson et al. (2024), respectively. On the other hand, In Methodology/other category are grouped articles related to pLCA development that do not fall into the first two categories (i.e. methodological papers).

For this reason, the articles classified as methodology/other, through a dedicated reading activity, are further subdivided into four sub-categories:

1. Guide/method for whole pLCA: papers proposing methods to develop completely a pLCA analysis.
2. Guide/method for part pLCA: papers describing methods for specific sections of pLCA (e.g., upscaling methods, background simulation methods).
3. Application of a methodology in case study: papers applying a specific method within a case study without performing a full pLCA analysis.
4. External/linked observation: remaining papers with related observations.

This categorization was designed to enable a thorough analysis of the available literature reviews and methodological papers, with the aim of constructing an updated framework for conducting pLCA based on the latest publications.

### Addressing Q1

In the first phase, only articles from the Literature review (12) and Methodology/other (38) categories were considered. Their contribution to the pLCA framework was assessed by assigning to each article one of the following labels:

- very important: when the information provided by the text are structural for the pLCA development; they have been used to answer to the following questions:
  - What a pLCA is?
  - When a pLCA can be defined?
  - How should a pLCA be implemented?
- important: Articles that has a key role in the development of the framework but need to be contextualized thanks the previous ones
- for a deeper knowledge: sectorial articles which develop a specific thematic useful for the framework

The articles reported in Table 2S have been used for the identification of the main methodological aspects already reported in the main document (Table 2 and schematized in Figure 3).

**Table S2**: Articles utilized for the identification of the main methodological aspects as answer to Q1: the firsts two columns report reference and title, the third and fourth columns contain the categories associated to each article, the last column show the assigned labels. ^[1–50]^

| **Reference** | **Title** | **Group** | **Subgroup** | **Label assigned** |
| --- | --- | --- | --- | --- |
| Karka et al., 2019 | Predictive LCA - a systems approach to integrate LCA decisions ahead of design | Literature Review |  | For a deeper knowledge |
| Buyle et al., 2019 | The Future of Ex-Ante LCA? Lessons Learned and Practical Recommendations | Literature Review |  | Very important |
| Hermansson et al., 2019 | Prospective study of lignin-based and recycled carbon fibers in composites through meta-analysis of life cycle assessments | Methodology/other | Guide/method PartPLCA | For a deeper knowledge |
| Van Der Giesen et al., 2020 | A critical view on the current application of LCA for new technologies and recommendations for improved practice | Literature Review |  | Important |
| Thonemann et al., 2020 | How to Conduct Prospective Life Cycle Assessment for Emerging Technologies? A Systematic Review and Methodological Guidance | Literature Review |  | Very important |
| Moni et al., 2020 | Life cycle assessment of emerging technologies: A review | Literature Review |  | Important |
| Bergerson et al., 2020 | Life cycle assessment of emerging technologies: Evaluation techniques at different stages of market and technical maturity | Literature Review |  | Very important |
| Tsoy et al., 2020 | Upscaling methods used in ex ante life cycle assessment of emerging technologies: a review | Literature Review |  | Very important |
| Van Der Hulst et al., 2020 | A systematic approach to assess the environmental impact of emerging technologies: A case study for the GHG footprint of CIGS solar photovoltaic laminate | Methodology/other | Guide/method wholePLCA | Very important |
| Mendoza Beltran et al., 2020 | When the Background Matters: Using Scenarios from Integrated Assessment Models in Prospective Life Cycle Assessment | Methodology/other | Guide/method PartPLCA | Very important |
| Cooper & Gutowski, 2020 | Prospective Environmental Analyses of Emerging Technology: A Critique, a Proposed Methodology, and a Case Study on Incremental Sheet Forming | Methodology/other | Guide/method PartPLCA | For a deeper knowledge |
| De Araújo E Silva et al., 2020 | An approach for implementing ecodesign at early research stage: A case study of bacterial cellulose production | Methodology/other | Guide/method PartPLCA | Important |
| Kawajiri et al., 2020 | Development of life cycle assessment of an emerging technology at research and development stage: A case study on single-wall carbon nanotube produced by super growth method | Methodology/other | Application_of_a_Methodology_in_case_study | For a deeper knowledge |
| Blanco et al., 2020 | Assessing the sustainability of emerging technologies: A probabilistic LCA method applied to advanced photovoltaics | Methodology/other | Guide/method PartPLCA | For a deeper knowledge |
| Bisinella et al., 2021 | Future scenarios and life cycle assessment: systematic review and recommendations | Literature Review |  | Important |
| Adrianto et al., 2021 | How can LCA include prospective elements to assess emerging technologies and system transitions? The 76th LCA Discussion Forum on Life Cycle Assessment, 19 November 2020 | Literature Review |  | For a deeper knowledge |
| Pizzol et al., 2021 | Non-linearity in the Life Cycle Assessment of Scalable and Emerging Technologies | Methodology/other | External/linked observation | For a deeper knowledge |
| Yousefzadeh & Lloyd, 2021 | Prospective life cycle assessment as a tool for environmentally responsible innovation | Methodology/other | Application_of_a_Methodology_in_case_study | For a deeper knowledge |
| Ferrero et al., 2021 | A PROBABILISTIC APPROACH FOR ESTIMATING THE ENVIRONMENTAL IMPACT OF NOVEL PRODUCT CONCEPTS | Methodology/other | External/linked observation | For a deeper knowledge |
| Pereira Da Silva et al., 2021 | Integrating life cycle assessment in early process development stage: The case of extracting starch from mango kernel | Methodology/other | Application_of_a_Methodology_in_case_study | For a deeper knowledge |
| Steubing & De Koning, 2021 | Making the use of scenarios in LCA easier: the superstructure approach | Methodology/other | Guide/method PartPLCA | Important |
| Hung et al., 2022 | ECOPT2: An adaptable life cycle assessment model for the environmentally constrained optimization of prospective technology transitions | Methodology/other | External/linked observation | For a deeper knowledge |
| Tsalidis & Korevaar, 2022 | Environmental assessments of scales: The effect of ex-ante and ex-post data on life cycle assessment of wood torrefaction | Methodology/other | External/linked observation | For a deeper knowledge |
| Sacchi et al., 2022 | PRospective EnvironMental Impact asSEment (premise): A streamlined approach to producing databases for prospective life cycle assessment using integrated assessment models | Methodology/other | Guide/method PartPLCA | Very important |
| Faber et al., 2022 | Adapting Technology Learning Curves for Prospective Techno-Economic and Life Cycle Assessments of Emerging Carbon Capture and Utilization Pathways | Methodology/other | Guide/method PartPLCA | Important |
| Ganesan & Valderrama, 2022 | Anticipatory life cycle analysis framework for sustainable management of end-of-life crystalline silicon photovoltaic panels | Methodology/other | Application_of_a_Methodology_in_case_study | For a deeper knowledge |
| Huppes & Schaubroeck, 2022 | Forecasting the Future Sustainability of Technology Choices: Qualitative Predictive Validity of Models as a Complement to Quantitative Uncertainty | Methodology/other | External/linked observation | For a deeper knowledge |
| Keller et al., 2022 | Life cycle inventory data generation by process simulation for conventional, feedstock recycling and power-to-X technologies for base chemical production | Methodology/other | Application_of_a_Methodology_in_case_study | For a deeper knowledge |
| Haase et al., 2022 | Prospective assessment of energy technologies: a comprehensive approach for sustainability assessment | Methodology/other | External/linked observation | For a deeper knowledge |
| Jouannais et al., 2022 | Stochastic LCA Model of Upscaling the Production of Microalgal Compounds | Methodology/other | Application_of_a_Methodology_in_case_study | For a deeper knowledge |
| Elginoz, Owusu-Agyeman, et al., 2022 | Application and adaptation of a scale-up framework for life cycle assessment to resource recovery from waste systems | Methodology/other | Application_of_a_Methodology_in_case_study | Important |
| Voglhuber-Slavinsky et al., 2022 | Setting life cycle assessment (LCA) in a future-oriented context: the combination of qualitative scenarios and LCA in the agri-food sector | Methodology/other | Guide/method PartPLCA | Important |
| Kikuchi & Kanematsu, 2022 | Application of CAPE Tools into Prospective Life Cycle Assessment: A Case Study in Acetylated Cellulose Nanofiber-Reinforced Plastics | Methodology/other | Application_of_a_Methodology_in_case_study | Important |
| Kikuchi et al., 2022 | Application of CAPE Tools into Prospective Life Cycle Assessment: A Case Study in Recycling Systems Design of Lithium-Ion Battery | Methodology/other | Application_of_a_Methodology_in_case_study | Important |
| Elginoz, Papadaskalopoulou, et al., 2022 | Using life cycle assessment at an early stage of design and development of zero discharge brine treatment and recovery | Methodology/other | External/linked observation | For a deeper knowledge |
| Steubing et al., 2023 | Conditions for the broad application of prospective life cycle inventory databases | Literature Review |  | Important |
| Spreafico et al., 2023 | A new method of patent analysis to support prospective life cycle assessment of eco-design solutions | Methodology/other | Guide/method PartPLCA | Important |
| Kikuchi et al., 2023 | Application of CAPE Tools into Prospective Life Cycle Assessment: A Case Study in Feedstock Recycling of Waste Plastics | Methodology/other | Application_of_a_Methodology_in_case_study | For a deeper knowledge |
| Sauve et al., 2023 | Integrated early-stage environmental and economic assessment of emerging technologies and its applicability to the case of plasma gasification | Methodology/other | Guide/method PartPLCA | For a deeper knowledge |
| Weyand et al., 2023 | Scheme for generating upscaling scenarios of emerging functional materials based energy technologies in prospective LCA (UpFunMatLCA) | Methodology/other | Guide/method PartPLCA | Very important |
| Fernández-González et al., 2023 | The Relevance of Life Cycle Assessment Tools in the Development of Emerging Decarbonization Technologies | Methodology/other | External/linked observation | For a deeper knowledge |
| Haupt et al., 2023 | Challenges of prospective life cycle assessment of emerging recycling processes: case study of battery materials recovery | Methodology/other | Guide/method PartPLCA | Important |
| Fayyaz et al., 2023 | Sustainable end -of-life value chain scenarios for wind turbine blades | Methodology/other | External/linked observation | For a deeper knowledge |
| Langkau et al., 2023 | A stepwise approach for Scenario-based Inventory Modelling for Prospective LCA (SIMPL) | Methodology/other | Guide/method PartPLCA | Very important |
| Erakca et al., 2024 | Systematic review of scale-up methods for prospective life cycle assessment of emerging technologies | Literature Review |  | Very important |
| Arvidsson et al., 2024 | Terminology for future-oriented life cycle assessment: review and recommendations | Literature Review |  | Very important |
| Arias et al., 2024 | Assessing the future prospects of emerging technologies for shipping and aviation biofuels: A critical review | Methodology/other | External/linked observation | For a deeper knowledge |
| Spreafico et al., 2024a | On the intersection between prospective LCA and patent analysis. A theoretical discussion | Methodology/other | Guide/method PartPLCA | Important |
| Spreafico et al., 2024b | Prospective Life Cycle Assessment Based on Patent Analysis to Support Eco-design | Methodology/other | Guide/method PartPLCA | Important |
| Kanematsu et al., 2024 | Prototyping cloud application for regional green transformation supported by prospective life cycle assessment | Methodology/other | External/linked observation | For a deeper knowledge |

### Addressing Q2

The identified methodological aspects have been then used, in the second phase, as benchmark for the evaluation of the 29 case studies’ structure, published from 2019-2024. In other terms, we compare the methodological development of all the case studies, assessing if the identified main aspects have been used by the respective authors, evaluating the completeness of the study. Depending on the number of criteria met and the methods implemented during the pLCA development, each case study has been classified as:

- Compatible: when almost all the identified Q1-methodological aspects were included into the case study
- Almost Compatible: when the background system has been not consistently upgraded according to the identified methodologies, while almost all the residual Q1-methodological aspects were included in the case study
- About Compatible: When the background system has not been upgraded, while almost all the residual Q1-methodological aspects were included in the case study
- Not Compatible: The same situation of About Compatible case, with in addition some imprecisions in the upscaling area

A summary of this classification based on the articles grouped as case study is reported in Table 3S, while the deeper comparison between the case studies published after 2021 and the identified Q1-methodological aspects is available in Table 2 of the main document.

**Table S3:** Articles classified as case study: the first two columns indicate the reference and the title respectively, while the last column contains the degree of comparability between each case study’s structure and the identified Q1-methodological aspects. ^[51–79]^

| **Reference** | **Title** | **Compatibility** |
| --- | --- | --- |
| Lu et al., 2019 | Deducing targets of emerging technologies based on ex ante life cycle thinking: Case study on a chlorine recovery process for polyvinyl chloride wastes | Not Compatible |
| Thonemann & Schulte, 2019 | From Laboratory to Industrial Scale: A Prospective LCA for Electrochemical Reduction of CO2 to Formic Acid | about compatible |
| Ambrose & Kendall, 2020 | Understanding the future of lithium: Part 2, temporally and spatially resolved life-cycle assessment modeling | Not Compatible |
| Grimaldi, Pucciarelli, et al., 2020 | Anticipatory life cycle assessment of gold nanoparticles production: Comparison of milli-continuous flow and batch synthesis | about compatible |
| Pallas et al., 2020 | Ex ante life cycle assessment of GaAs/Si nanowire-based tandem solar cells: a benchmark for industrialization | about compatible |
| Elginoz et al., 2020 | Ex-ante life cycle assessment of volatile fatty acid production from dairy wastewater | about compatible |
| Grimaldi, De Leon Izeppi, et al., 2020 | Life cycle assessment and cost evaluation of emerging technologies at early stages: The case of continuous flow synthesis of Rufinamide | Not Compatible |
| Yao et al., 2020 | Life cycle assessment of 3D printing geo-polymer concrete: An ex-ante study | about compatible |
| Bartolozzi et al., 2020 | Life cycle assessment of emerging environmental technologies in the early stage of development: A case study on nanostructured materials | about compatible |
| Lu et al., 2021 | Sustainable Advance of Cl Recovery from Polyvinyl Chloride Waste Based on Experiment, Simulation, and Ex Ante Life-Cycle Assessment | about compatible |
| Delpierre et al., 2021 | Assessing the environmental impacts of wind-based hydrogen production in the Netherlands using ex-ante LCA and scenarios analysis | almost compatible |
| Maes et al., 2021 | Enhanced fly ash use in concrete: Ex-ante LCA on an emerging electro-mass separation technology | almost compatible |
| Winter et al., 2021 | Towards aromatics from biomass: Prospective Life Cycle Assessment of bio-based aniline | Not Compatible |
| Carlqvist et al., 2022 | Life cycle assessment for identification of critical aspects in emerging technologies for the extraction of phenolic compounds from spruce bark | about compatible |
| Adrianto & Pfister, 2022 | Prospective environmental assessment of reprocessing and valorization alternatives for sulfidic copper tailings | about compatible |
| García-Cruz et al., 2022 | Prospective life cycle assessment of a based orange wax fungicide | about compatible |
| Zhang et al., 2022 | Prospective life cycle assessment of a flexible all-organic battery | almost compatible |
| Calero et al., 2022 | Upscaling via a Prospective LCA: A Case Study on Tomato Homogenate Using a Near-to-Market Pasteurisation Technology | almost compatible |
| Lai et al., 2022 | Employing a Socio-Technical System Approach in Prospective Life Cycle Assessment: A Case of Large-Scale Swedish Sustainable Aviation Fuels | compatible |
| Sander-Titgemeyer et al., 2023 | Applying an iterative prospective LCA approach to emerging wood-based technologies: three German case studies | compatible |
| Eltohamy et al., 2023 | Ex-ante life cycle assessment of FineFuture flotation technology: case study of Grecian Magnesite | almost compatible |
| Fuentes et al., 2023 | Life Cycle Assessment of Magnetite Production Using Microfluidic Devices: Moving from the Laboratory to Industrial Scale | about compatible |
| Schulte et al., 2023 | Prospective LCA of Waste Electrical and Electronic Equipment Thermo-Chemical Recycling by Pyrolysis | about compatible |
| Kamali et al., 2023 | Prospective Life Cycle Assessment of Two Supercapacitor Architectures | about compatible |
| Van Der Hulst et al., 2024 | Comparing Environmental Impacts of Single-Junction Silicon and Silicon/Perovskite Tandem Photovoltaics-A Prospective Life Cycle Assessment | compatible |
| Santiago-Herrera et al., 2024 | Ex-ante life cycle assessment of directed energy deposition based additive manufacturing: A comparative gearbox production case study | about compatible |
| Heberl et al., 2024 | Prospective Life Cycle Assessment of Biological Methanation in a Trickle-Bed Pilot Plant and a Potential Scale-Up | almost compatible |
| Kamali et al., 2024 | Steering Innovation toward Sustainable Electrochromic Displays: A Prospective Life Cycle Assessment Study | almost compatible |
| Accardo et al., 2024 | Prospective LCA of Next-Generation Cells for Electric Vehicle Applications | almost compatible |

## References

[1] L. R. Adrianto, M. K. Van Der Hulst, J. P. Tokaya, R. Arvidsson, C. F. Blanco, C. Caldeira, G. Guillén-Gonsálbez, S. Sala, B. Steubing, M. Buyle, M. Kaddoura, N. H. Navarre, J. Pedneault, M. Pizzol, B. Salieri, T. Van Harmelen, M. Hauck, *Int J Life Cycle Assess* **2021**, *26*, 1541–1544.

[2] A. Arias, C.-E. Nika, V. Vasilaki, G. Feijoo, M. T. Moreira, E. Katsou, *Renewable and Sustainable Energy Reviews* **2024**, *197*, 114427.

[3] R. Arvidsson, M. Svanström, B. A. Sandén, N. Thonemann, B. Steubing, S. Cucurachi, *Int J Life Cycle Assess* **2024**, *29*, 607–613.

[4] J. A. Bergerson, A. Brandt, J. Cresko, M. Carbajales‐Dale, H. L. MacLean, H. S. Matthews, S. McCoy, M. McManus, S. A. Miller, W. R. Morrow, I. D. Posen, T. Seager, T. Skone, S. Sleep, *J of Industrial Ecology* **2020**, *24*, 11–25.

[5] V. Bisinella, T. H. Christensen, T. F. Astrup, *Int J Life Cycle Assess* **2021**, *26*, 2143–2170.

[6] C. F. Blanco, S. Cucurachi, J. B. Guinée, M. G. Vijver, W. J. G. M. Peijnenburg, R. Trattnig, R. Heijungs, *Journal of Cleaner Production* **2020**, *259*, 120968.

[7] M. Buyle, A. Audenaert, P. Billen, K. Boonen, S. Van Passel, *Sustainability* **2019**, *11*, 5456.

[8] D. R. Cooper, T. G. Gutowski, *J of Industrial Ecology* **2020**, *24*, 38–51.

[9] R. De Araújo E Silva, A. I. Santa Brígida, M. De Freitas Rosa, R. M. Da Silva Neto, W. A. Spinosa, E. Benício De Sá Filho, M. C. Brito De Figueirêdo, *Journal of Cleaner Production* **2020**, *269*, 122245.

[10] N. Elginoz, I. Owusu-Agyeman, G. Finnveden, R. Hischier, T. Rydberg, Z. Cetecioglu, *Journal of Cleaner Production* **2022**, *355*, 131720.

[11] N. Elginoz, C. Papadaskalopoulou, S. Harris, *Water Resources and Industry* **2022**, *28*, 100184.

[12] M. Erakca, M. Baumann, C. Helbig, M. Weil, *Journal of Cleaner Production* **2024**, *451*, 142161.

[13] G. Faber, A. Ruttinger, T. Strunge, T. Langhorst, A. Zimmermann, M. Van Der Hulst, F. Bensebaa, S. Moni, L. Tao, *Front. Clim.* **2022**, *4*, 820261.

[14] S. Fayyaz, K. W. Lund, B. Khoshnevisan, E. S. Madsen, M. Birkved, *J. Phys.: Conf. Ser.* **2023**, *2507*, 012007.

[15] J. Fernández-González, M. Rumayor, A. Domínguez-Ramos, A. Irabien, I. Ortiz, *JACS Au* **2023**, *3*, 2631–2639.

[16] V. Ferrero, C. Hoyle, B. DuPont, in *Volume 3B: 47th Design Automation Conference (DAC)*, American Society Of Mechanical Engineers, Virtual, Online, **2021**, p. V03BT03A046.

[17] K. Ganesan, C. Valderrama, *Energy* **2022**, *245*, 123207.

[18] M. Haase, C. Wulf, M. Baumann, C. Rösch, M. Weil, P. Zapp, T. Naegler, *Energ Sustain Soc* **2022**, *12*, 20.

[19] J. Haupt, N. Kononova, F. Cerdas, S. Zellmer, C. Herrmann, *Procedia CIRP* **2023**, *116*, 23–28.

[20] F. Hermansson, M. Janssen, M. Svanström, *Journal of Cleaner Production* **2019**, *223*, 946–956.

[21] C. R. Hung, P. Kishimoto, V. Krey, A. H. Strømman, G. Majeau‐Bettez, *J of Industrial Ecology* **2022**, *26*, 1616–1630.

[22] G. Huppes, T. Schaubroeck, *Front. Sustain.* **2022**, *3*, 629653.

[23] P. Jouannais, S. Hindersin, S. Löhn, M. Pizzol, *Environ. Sci. Technol.* **2022**, *56*, 10454–10464.

[24] Y. Kanematsu, S. Fujii, Y. Oshita, S. Ohara, A. Komori, D. Shimotoku, K. Iizuka, J. Kawase, H. Kobayashi, Y. Kikuchi, in *Computer Aided Chemical Engineering*, Elsevier, **2024**, pp. 3463–3468.

[25] P. Karka, S. Papadokonstantakis, A. Kokossis, in *Computer Aided Chemical Engineering*, Elsevier, **2019**, pp. 97–102.

[26] K. Kawajiri, T. Goto, S. Sakurai, K. Hata, K. Tahara, *Journal of Cleaner Production* **2020**, *255*, 120015.

[27] F. Keller, P. Mamani Soliz, L. G. Seidl, R. P. Lee, B. Meyer, *Data in Brief* **2022**, *41*, 107848.

[28] Y. Kikuchi, A. Heiho, Y. Dou, I. Suwa, C. Tokoro, in *Computer Aided Chemical Engineering*, Elsevier, **2022**, pp. 1585–1590.

[29] Y. Kikuchi, Y. Kanematsu, in *Computer Aided Chemical Engineering*, Elsevier, **2022**, pp. 1867–1872.

[30] Y. Kikuchi, Y. Nomura, T. Nakamura, S. Fujii, A. Heiho, Y. Kanematsu, in *Computer Aided Chemical Engineering*, Elsevier, **2023**, pp. 2477–2482.

[31] S. Langkau, B. Steubing, C. Mutel, M. P. Ajie, L. Erdmann, A. Voglhuber-Slavinsky, M. Janssen, *Int J Life Cycle Assess* **2023**, *28*, 1169–1193.

[32] A. Mendoza Beltran, B. Cox, C. Mutel, D. P. Van Vuuren, D. Font Vivanco, S. Deetman, O. Y. Edelenbosch, J. Guinée, A. Tukker, *J of Industrial Ecology* **2020**, *24*, 64–79.

[33] S. M. Moni, R. Mahmud, K. High, M. Carbajales‐Dale, *J of Industrial Ecology* **2020**, *24*, 52–63.

[34] A. K. Pereira Da Silva, A. Cardoso, E. Benício De Sá Filho, H. Monteiro Cordeiro De Azeredo, F. Freire, F. Casimiro Filho, M. C. Brito De Figueirêdo, *Journal of Cleaner Production* **2021**, *321*, 128981.

[35] M. Pizzol, R. Sacchi, S. Köhler, A. Anderson Erjavec, *Front. Sustain.* **2021**, *1*, 611593.

[36] R. Sacchi, T. Terlouw, K. Siala, A. Dirnaichner, C. Bauer, B. Cox, C. Mutel, V. Daioglou, G. Luderer, *Renewable and Sustainable Energy Reviews* **2022**, *160*, 112311.

[37] G. Sauve, J. L. Esguerra, D. Laner, J. Johansson, N. Svensson, S. Van Passel, K. Van Acker, *Journal of Cleaner Production* **2023**, *382*, 134684.

[38] C. Spreafico, D. Landi, D. Russo, *Sustainable Production and Consumption* **2023**, *38*, 241–251.

[39] C. Spreafico, D. Landi, D. Russo, *Procedia CIRP* **2024**, *122*, 211–216.

[40] C. Spreafico, D. Landi, D. Russo, in *Design Tools and Methods in Industrial Engineering III* (Eds.: M. Carfagni, R. Furferi, P. Di Stefano, L. Governi, F. Gherardini), Springer Nature Switzerland, Cham, **2024**, pp. 331–338.

[41] B. Steubing, D. De Koning, *Int J Life Cycle Assess* **2021**, *26*, 2248–2262.

[42] B. Steubing, A. Mendoza Beltran, R. Sacchi, *Int J Life Cycle Assess* **2023**, *28*, 1092–1103.

[43] N. Thonemann, A. Schulte, D. Maga, *Sustainability* **2020**, *12*, 1192.

[44] G. A. Tsalidis, G. Korevaar, *Resources, Conservation and Recycling* **2022**, *176*, 105906.

[45] N. Tsoy, B. Steubing, C. Van Der Giesen, J. Guinée, *Int J Life Cycle Assess* **2020**, *25*, 1680–1692.

[46] C. Van Der Giesen, S. Cucurachi, J. Guinée, G. J. Kramer, A. Tukker, *Journal of Cleaner Production* **2020**, *259*, 120904.

[47] M. K. Van Der Hulst, M. A. J. Huijbregts, N. Van Loon, M. Theelen, L. Kootstra, J. D. Bergesen, M. Hauck, *J of Industrial Ecology* **2020**, *24*, 1234–1249.

[48] A. Voglhuber-Slavinsky, A. Zicari, S. Smetana, B. Moller, E. Dönitz, L. Vranken, M. Zdravkovic, K. Aganovic, E. Bahrs, *Eur J Futures Res* **2022**, *10*, 15.

[49] S. Weyand, K. Kawajiri, C. Mortan, L. Schebek, *J of Industrial Ecology* **2023**, *27*, 676–692.

[50] Z. Yousefzadeh, S. M. Lloyd, in *2021 IEEE International Symposium on Technology and Society (ISTAS)*, IEEE, Waterloo, ON, Canada, **2021**, pp. 1–9.

[51] A. Accardo, A. Garofalo, G. Dotelli, E. Spessa, *IEEE Access* **2024**, *12*, 19584–19597.

[52] L. R. Adrianto, S. Pfister, *Resources, Conservation and Recycling* **2022**, *186*, 106567.

[53] H. Ambrose, A. Kendall, *J of Industrial Ecology* **2020**, *24*, 90–100.

[54] I. Bartolozzi, T. Daddi, C. Punta, A. Fiorati, F. Iraldo, *J of Industrial Ecology* **2020**, *24*, 101–115.

[55] M. Calero, G. Clemente, D. Fartdinov, S. Bañón, I. Muñoz, N. Sanjuán, *Sustainability* **2022**, *14*, 1716.

[56] K. Carlqvist, O. Wallberg, G. Lidén, P. Börjesson, *Journal of Cleaner Production* **2022**, *333*, 130093.

[57] M. Delpierre, J. Quist, J. Mertens, A. Prieur-Vernat, S. Cucurachi, *Journal of Cleaner Production* **2021**, *299*, 126866.

[58] N. Elginoz, M. Atasoy, G. Finnveden, Z. Cetecioglu, *Journal of Cleaner Production* **2020**, *269*, 122267.

[59] H. Eltohamy, G. Cecere, L. Rigamonti, *Int J Life Cycle Assess* **2023**, *28*, 1348–1365.

[60] O. P. Fuentes, J. C. Cruz, E. Mignard, G. Sonnemann, J. F. Osma, *ACS Sustainable Chem. Eng.* **2023**, *11*, 6932–6943.

[61] A. García-Cruz, L. Díaz-Jiménez, A. Ilyina, S. Carlos-Hernández, *Industrial Crops and Products* **2022**, *180*, 114769.

[62] F. Grimaldi, G. A. De Leon Izeppi, D. Kirschneck, P. Lettieri, M. Escribà‐Gelonch, V. Hessel, *J Adv Manuf & Process* **2020**, *2*, e10043.

[63] F. Grimaldi, M. Pucciarelli, A. Gavriilidis, P. Dobson, P. Lettieri, *Journal of Cleaner Production* **2020**, *269*, 122335.

[64] M. Heberl, C. Withelm, A. Kaul, D. Rank, M. Sterner, *Energies* **2024**, *17*, 2206.

[65] A. K. Kamali, E. Glogic, N. M. Keppetipola, G. Sonnemann, T. Toupance, L. Cojocaru, *ACS Sustainable Chem. Eng.* **2023**, *11*, 15898–15909.

[66] A. K. Kamali, D. Lee, R. Futsch, E. Glogic, A. Rougier, G. Sonnemann, *ACS Sustainable Chem. Eng.* **2024**, *12*, 1501–1513.

[67] Y. Y. Lai, E. Karakaya, A. Björklund, *Front. Sustain.* **2022**, *3*, 912676.

[68] J. Lu, S. Kumagai, Y. Fukushima, H. Ohno, S. Borjigin, T. Kameda, Y. Saito, T. Yoshioka, *ACS Sustainable Chem. Eng.* **2021**, *9*, 14112–14123.

[69] J. Lu, S. Kumagai, H. Ohno, T. Kameda, Y. Saito, T. Yoshioka, Y. Fukushima, *Resources, Conservation and Recycling* **2019**, *151*, 104500.

[70] B. Maes, M. Buyle, A. Audenaert, B. Craeye, *Cleaner Engineering and Technology* **2021**, *2*, 100076.

[71] G. Pallas, M. G. Vijver, W. J. G. M. Peijnenburg, J. Guinée, *Int J Life Cycle Assess* **2020**, *25*, 1767–1782.

[72] A. Sander-Titgemeyer, M. Risse, G. Weber-Blaschke, *Int J Life Cycle Assess* **2023**, *28*, 495–515.

[73] M. Santiago-Herrera, E. Igos, J. M. Alegre, S. Martel-Martín, R. Barros, *Sustainable Materials and Technologies* **2024**, *39*, e00819.

[74] A. Schulte, M. Lamb-Scheffler, P. Biessey, T. Rieger, *Chemie Ingenieur Technik* **2023**, *95*, 1268–1281.

[75] N. Thonemann, A. Schulte, *Environ. Sci. Technol.* **2019**, *53*, 12320–12329.

[76] M. K. Van Der Hulst, D. Magoss, Y. Massop, S. Veenstra, N. Van Loon, I. Dogan, G. Coletti, M. Theelen, S. Hoeks, M. A. J. Huijbregts, R. Van Zelm, M. Hauck, *ACS Sustainable Chem. Eng.* **2024**, *12*, 8860–8870.

[77] B. Winter, R. Meys, A. Bardow, *Journal of Cleaner Production* **2021**, *290*, 125818.

[78] Y. Yao, M. Hu, F. Di Maio, S. Cucurachi, *J of Industrial Ecology* **2020**, *24*, 116–127.

[79] S. Zhang, N. Ericsson, M. Sjödin, H. Karlsson Potter, P.-A. Hansson, Å. Nordberg, *Journal of Cleaner Production* **2022**, *373*, 133804.
